# BMC pregnancy and childbirth - ‘screening and management of food insecurity in pregnancy’

**DOI:** 10.1186/s12884-023-06062-x

**Published:** 2023-12-15

**Authors:** Kingsley Emwinyore Agho, Paige van der Pligt

**Affiliations:** 1 School of Health Sciences, Western Sydney University, Campbelltown, NSW 2560 Australia; 2 Translational Health Research Institute (THRI), Western Sydney University, Campbelltown, NSW 2560 Australia; 3 African Vision Research Institute (AVRI), University of KwaZulu-Natal, Westville Campus, Durban, 3629 South Africa; 4 Institute for Physical Activity and Nutrition (IPAN), School of Exercise and Nutrition Sciences, Deakin University, Geelong, 3220 Australia; 5 Department of Nutrition and Dietetics, Western Health, Footscray, Australia

## Abstract

Addressing food insecurity during pregnancy is a major public health problem that demands guided interventions and translational research in public health. In this Editorial, we provide the context and invite contributions for our *BMC Pregnancy and Childbirth* Collection on Screening and management of food insecurity in pregnancy.

## Main text

Food security exists when people have social, physical, and economic access to sufficient, safe, and nutritious food to meet their needs and dietary preferences to sustain a healthy life. Food insecurity, with hunger or without hunger, is a major public health issue. Evidence from the 2022 United Nations Food and Agriculture Organization (FAO) report indicated that over one in ten individuals worldwide (11.7%) suffered from severe food insecurity, corresponding to about 924 million individuals [1, 2]. These estimates reported by FAO for severe food insecurity are expected to increase significantly in the next few years due to regional armed conflict, war, climate change, and global economic hardship [3]. Food insecurity may have been further aggravated globally due to the recent COVID-19 pandemic [4, 5].

Pregnant women are especially vulnerable to food insecurity and its negative impacts, as they have increased nutritional requirements to meet the nutrient demands of the growing baby [6, 7]. Complex evidence from clinical research and public health literature has noted that many pregnant women who experience food insecurity are single women with children, women with a disabled parent or child, women who currently or previously lived with someone incarcerated, and pregnant women with grandchildren present and who smoke [8, 9]. Emerging evidence suggests that pregnant women experiencing food insecurity may also be at risk for domestic violence for multiple and complex reasons.

Food insecurity is associated with multiple adverse health outcomes for the mother and baby, including antenatal depression and anxiety, gestational diabetes, inadequate or excess gestational weight gain, anaemia and pregnancy-induced hypertension. Babies born to mothers who are food insecure are at higher risk for low birth weight, preterm birth, and developmental problems. Food insecurity during pregnancy can exacerbate postnatal stress and be associated with reduced quality of life and psychosocial outcomes, including increased postnatal depression and anxiety [9, 10]. Food insecurity and hunger among pregnant women can also have lasting health effects beyond the immediate postnatal period.

Given these serious effects of food insecurity during pregnancy, addressing it is crucial. Doing so will support the achievement of the Sustainable Development Goals (SDGs), especially SDG 2: Zero Hunger, SDG 3: Good Health and Well-being, and SDG 5: Gender equality. Understanding that food insecurity is a key factor that threatens pregnant women is vital in supporting the short- and long-term health of pregnant women and their unborn babies.

Currently, there is a lack of effective solutions to address hunger and food insecurity during pregnancy. A systematic review of 11 studies [6], mainly conducted in low- and middle-income countries, has identified various interventions to address these issues. The review concluded that robust evaluations of these interventions are lacking and may need to be more widely generalisable. In addition, there is a need for more research on long-term interventions.

Furthermore, the practice of antenatal screening for food insecurity has yet to be widely implemented in antenatal healthcare settings globally. It is crucial that we find ways to effectively screen and manage pregnant women who are at risk of food insecurity or who are food insecure. The development of models of care that address this issue is urgently needed to ensure the health and well-being of both mothers and their unborn babies. We must prioritise implementing these models to better support those who are the most vulnerable pregnant women in our communities.

Tackling food insecurity during pregnancy is a crucial health issue often overlooked in many countries worldwide. Prioritising the health and development challenges faced by pregnant women experiencing food insecurity is crucial for public health intervention research since we are now only seven years away from the SDG target year of 2030. Public health researchers need to take action to ensure that pregnant women have access to the necessary resources to maintain a healthy diet and protect the health of both themselves and their unborn children. Hence, more high-level research and efforts are needed if we are to continue to make gains in this research area and realise the full potential of food security for pregnant women and their babies. This Collection invites submissions on the use of novel methods to screen and manage food-insecure pregnant women and research exploring the short and long-term health benefits of food security among pregnant women and their unborn babies.

## Data Availability

n/a.

## Abbreviations

SDG: Sustainable Development Goal
FAO: Food and Agriculture Organization
COVID-19: Coronavirus disease
